# Towards inventory control excellence: An innovative approach based on a web-based platform

**DOI:** 10.12688/f1000research.140745.1

**Published:** 2023-11-15

**Authors:** Miguel Ramos-Miller, Alex Pacheco

**Affiliations:** 1 Faculty of Engineering, Universidad Nacional de Cañete, Cañete, Lima, 15701, Peru

**Keywords:** Inventory, system, web system, inventory control

## Abstract

**Background:**

Inventory management in educational institutions presents unique challenges due to the diverse sources of information and the need for improved team collaboration. This research aims to enhance inventory processes in educational institutions by leveraging information technology to optimize efficiency and ensure data integrity and reliability.

**Methods:**

The study employed a five-phase methodology to develop a web-based inventory optimization system. The initial phase involved a comprehensive analysis of inventory requirements, considering multiple data sources and team needs. Subsequently, in the planning phase, requirements were prioritized, and tasks with corresponding deadlines were established.

The implementation phase adhered to the requirements outlined in the planning phase, utilizing appropriate information technologies to ensure efficient and reliable operation. A thorough system review was conducted to assess the achievement of each requirement’s objectives, with necessary adjustments made as needed. Finally, following the completion of the previous phases, the developed software was deployed, and additional testing was conducted to ensure proper functionality.

**Results:**

Following the implementation of the web-based system, significant improvements were observed: an 85.51% increase in efficiency for goods searches, streamlining the process and reducing location times; a 90.31% enhancement in goods registration, ensuring greater accuracy and data reliability; an 83.11% improvement in annual goods report generation, simplifying reporting and offering a clearer view of the inventory. Overall, the inventory process experienced an 86.31% improvement, leading to enhanced efficiency and collaboration among administrative and teaching staff. The utilization of information technology reduced inventory processing times and ensured the uniqueness and reliability of information.

**Conclusions:**

This research focuses specifically on optimizing inventory management in educational institutions through information technology. The study’s uniqueness lies in its tailored web-based system designed to address the specific needs of educational institutions. The results demonstrate the effectiveness of this approach and its positive impact on inventory management.

## Introduction

Over the past decade, there has been a significant surge in the popularity of web-based systems worldwide, primarily attributed to their intricate nature and ability to facilitate simultaneous user support ( Mora, 2011). A notable example is the study by Qin *et al.* (2021), which implemented a system for reagent chemicals to facilitate searches and instantly know their location, allowing scientists to focus on developing new drugs. Research by Chila and Susi (2019) in the United States also highlighted the importance of information technology (IT) systems in inventory management, finding that supply management was time consuming in interventional radiology patient care. Using Lean principles, which refer to a set of practices and concepts designed to optimize efficiency and eliminate waste in business processes ( Kumar *et al.*, 2022), evaluated and improved an outdated process and implemented an inventory control system that resulted in significant time and cost savings.

One of the most prominent types of systems are web systems or applications. According to Molina-Ríos and Pedreira-Souto (2020), these systems are similar to traditional software and require development processes that include requirements gathering and programming in different languages, which can lead to heterogeneity during their development. In addition, the widespread adoption of web systems, driven by their rapid growth and numerous advantages, has significantly impacted e-commerce ( Castilla *et al.*, 2020). These systems facilitate direct interaction between end-users and the content of web pages. In addition, research in the UK has developed a machine learning-based computer system that can perform quality control by identifying defective parts and stopping the production process ( Papananias *et al.*, 2020).

**Figure 1.**
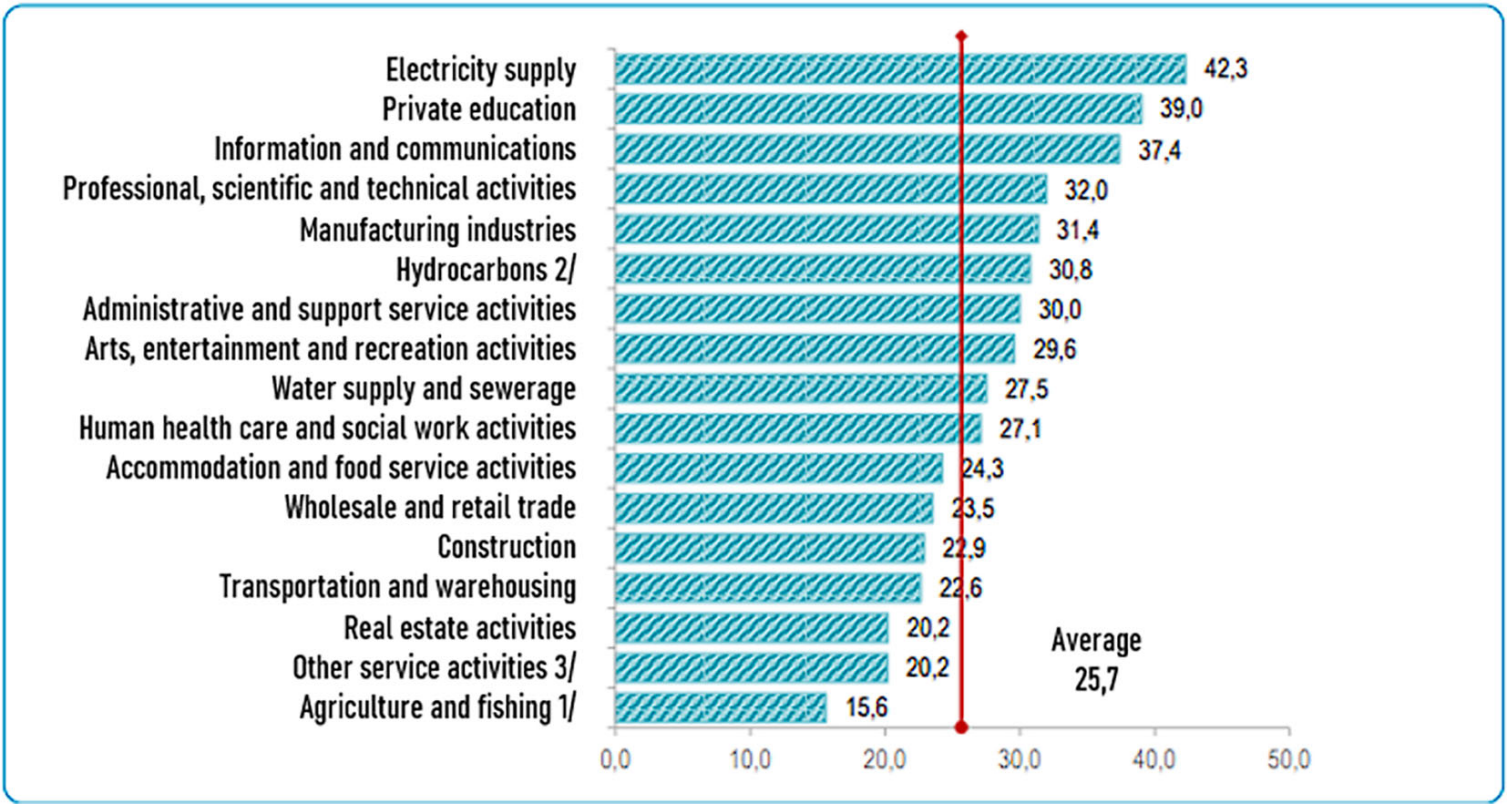
Companies that invest in science and technology, by economic activity. Note: Percentage of investment in science and technology by company according to economic activity. Source: ( Instituto Nacional de Estadística e Informática, 2019) – Reproduced with permission under the terms of the Instituto Nacional de Estadística e Informática's open licensing policy.

In the South American country of Peru, many public and private organizations have implemented software to automate their processes ( Centrum-PUCP, 2023). This gives them a competitive advantage by allowing them to save resources in terms of money, time and personnel ( Wilson & Mergel, 2022). In this regard, Figure 1 shows the sectors of companies that have invested the most in technology, highlighting the following “electricity (42.3%), private education (39.0%), information and communication (37.4%), professional, scientific and technical activities (32.0%), manufacturing (31.4%) and hydrocarbons (30.8%), among the most important” ( Instituto Nacional de Estadística e Informática, 2019).

In the public education sector, entities are responsible for accounting, controlling and reporting their goods in a responsible manner. These educational institutions are subordinated to the Local Education Management Unit (UGEL), as an example of decentralisation of local government, and depend on the Regional Directorate of Education (DRE) for administrative, normative and technical support ( D. S. No 019-2019-VIVIENDA, 2019). Management follows the rules of the National Superintendence of State Goods (SBN), which standardises codes and processes for each public good. Failure or delay in sending this information may result in a call for attention, a memorandum or the withholding of economic resources.

In Ancash, Peru, a web-based inventory management system was implemented in UGEL Aija, resulting in a 43.15% improvement in the supply rate and a 36.3% increase in the inventory turnover rate ( Sena, 2021). On the other hand, in Piura with Chiroque (2018), an inventory system was developed and the average times for registering goods, generating reports, searching for an good and ordering an good were improved. Similarly, Ramos (2016) implemented a system in the Telematics Office of the Police Front of Puno, Peru, using the agile methodology Extreme Programming (XP), which, thanks to its flexibility, allowed iterative acceleration and achieved functionalities such as improving the inventory work by 60% of the total.

Currently, according to information provided by officials at public educational institutions in San Vicente de Cañete, Lima, Peru, such as the José Buenaventura Sepúlveda Public Educational Institution, good information is recorded on physical documents and then transcribed into Excel spreadsheets. This has reportedly resulted in disorganized and duplicated records throughout the year, as well as the generation of reports with incorrect or outdated data. Therefore, the aim of this research is to implement web-based software that will improve the inventory control process and provide the institution with a technological advance to meet new educational challenges. In addition, it aims to provide accurate information about the goods, which will allow efficient management of the institution’s resources and indirectly promote quality education for both students and society.

While it is true that there are several applications with similar purposes in other sectors ( Pereira *et al.,* 2022), it is important to note that the application described in this article has been developed specifically for the education sector, taking into account the particular needs and requirements of an institution that lacks both technological tools and knowledge in the implementation of technology in educational processes. In addition, the application complies with the laws and regulations of the education sector regarding information management. ( Chiroque, 2018). It is important to highlight that this tool is innovative in the educational context and represents an important solution to improve the inventory control process of educational institutions. In particular, the application demonstrates how technology can be used to improve public services and promote digital transformation ( Castilla *et al.*, 2023). This may be relevant for other institutions that wish to implement digital solutions to improve information management ( Ramos, 2016). It therefore represents an innovation in the local context and contributes to the advancement of the implementation of digital solutions in the education sector.

## Methods

In this section, we provide a detailed account of the methods employed in the development and operation of our software tool tailored for educational institution inventory control.

### Implementation

Development Technologies: Our software tool was meticulously crafted using a combination of cutting-edge technologies. The backend is powered by Laravel 9, an open-source PHP framework known for its robust feature, leveraging the robust features of PHP 8.1, while the frontend is built on Vue3, an open-source Javascript framrwork that ensuresing a dynamic and responsive user interface.

Strategic Plug-in Integration: To augment the functionality of our software tool, we judiciously integrated several essential plug-ins and libraries, including:

• “laravel-dompdf” for PDF report generation.
• “laravel/sanctum” to fortify application programming interface (API) authentication.
• “maatwebsite/excel” for advanced Excel export capabilities.

Customization for educational institutions: The core framework of our software was customized to align with the unique requirements of educational institutions. This involved tailoring good categorization, user roles, and reporting functionalities to cater specifically to the needs of educational inventory management.

### Operation

Minimal system requirements: Our web-based inventory control system operates smoothly with minimal system prerequisites. These include:

• Server:
  ○ A server environment compatible with Laravel 9.
  ○ PHP 8.1 support.
  ○ Adequate storage capacity for housing inventory data.
• Client:
  ○ A modern web browser with JavaScript enabled.
  ○ Internet connectivity for web-based access.

By adhering to these minimal system requirements, we ensure that our software tool remains accessible and functional, even in resource-constrained educational settings.

### Unique features

This software tool boasts distinctive features that set it apart from existing solutions:

• Educational institution-centric: our software is purpose-built for educational institutions, accommodating their specific workflows and requirements for efficient inventory management.
• Tailored customization: users can seamlessly customize workflows, good categorization, and reporting features to suit their institution’s needs, making it a versatile solution.
• Enhanced functionality: intergration of essential plug-ins and libraries enhances the software’s capabilities, ensuring comprehensive inventory management.

By outlining these methods and unique features, we provide a clear blueprint for the development and implementation of our software tool within educational institutions, enhancing its replicability and utility.

## Use cases

Preface: Data Initialization

The system data is loaded automatically following the installation steps detailed in the “Readme.md” file found in the repository ( Ramos-Miller (b), 2023).

### Use case 1: goods search

**Figure 2.**
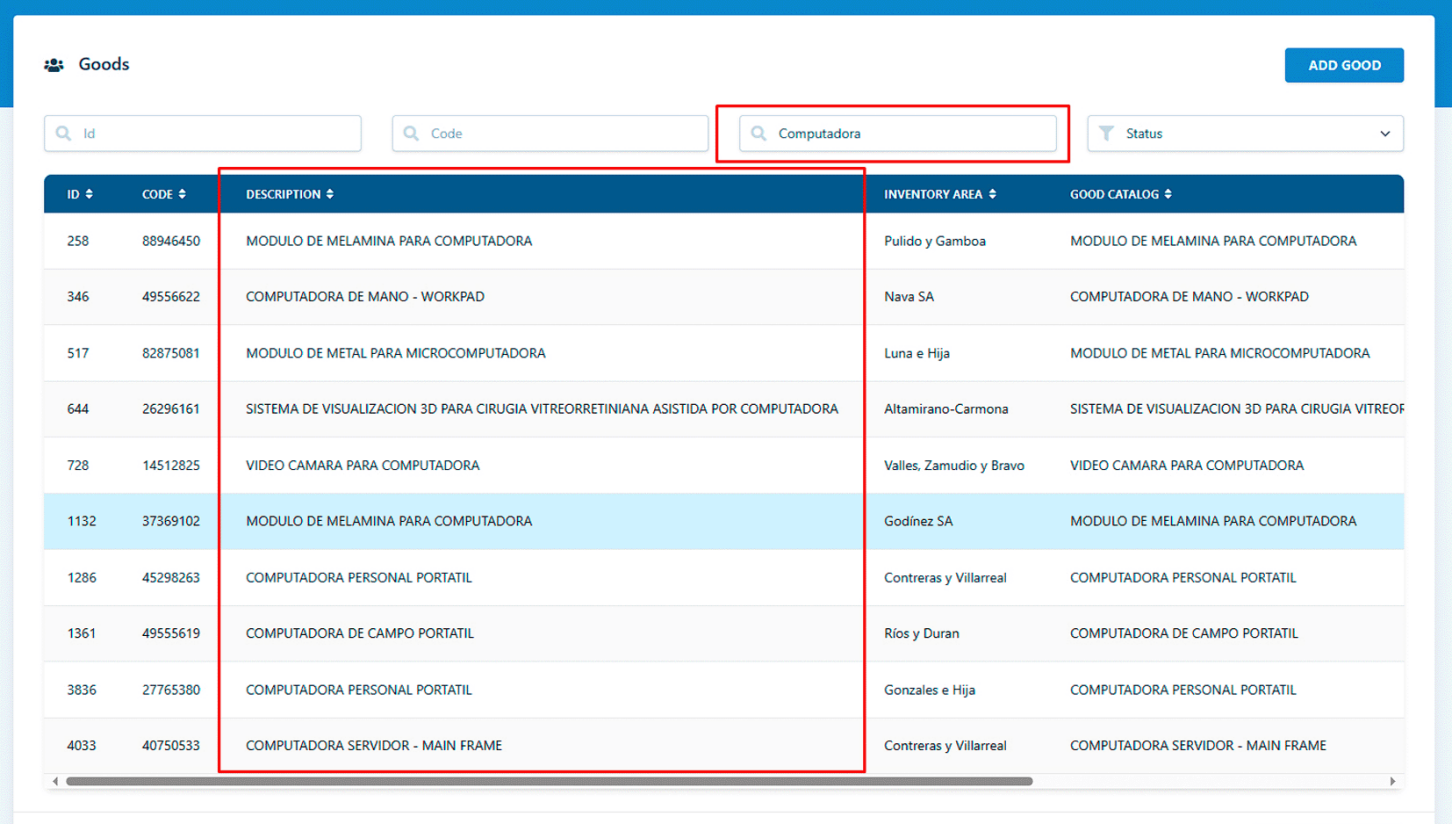
Goods search.

To demonstrate the software’s functionality, we present a specific use case involving the search for goods within the educational institution’s inventory. In this scenario, a user initiates a goods search by specifying criteria such as the item’s name, category, location, or status. Upon submission, the software retrieves and displays a list of matching goods based on the provided criteria. This practical example illustrates the software’s effectiveness in helping staff quickly locate specific goods within the institution’s inventory, as show in Figure 2.

**Input:** Search input query: “Computadora”

**Output:** List of coincidences:

• MODULO DE MELAMINA PARA COMPUTADORA
  ○ Description, Status, Inventory Area, etc …
• COMPUTADORA DE MANO - WORKPAD
  ○ Description, Status, Inventory Area, etc …
• MODULO DE METAL PARA MICROCOMPUTADORA
  ○ Description, Status, Inventory Area, etc …

### Use case2: Statistical reports

**Figure 3.**
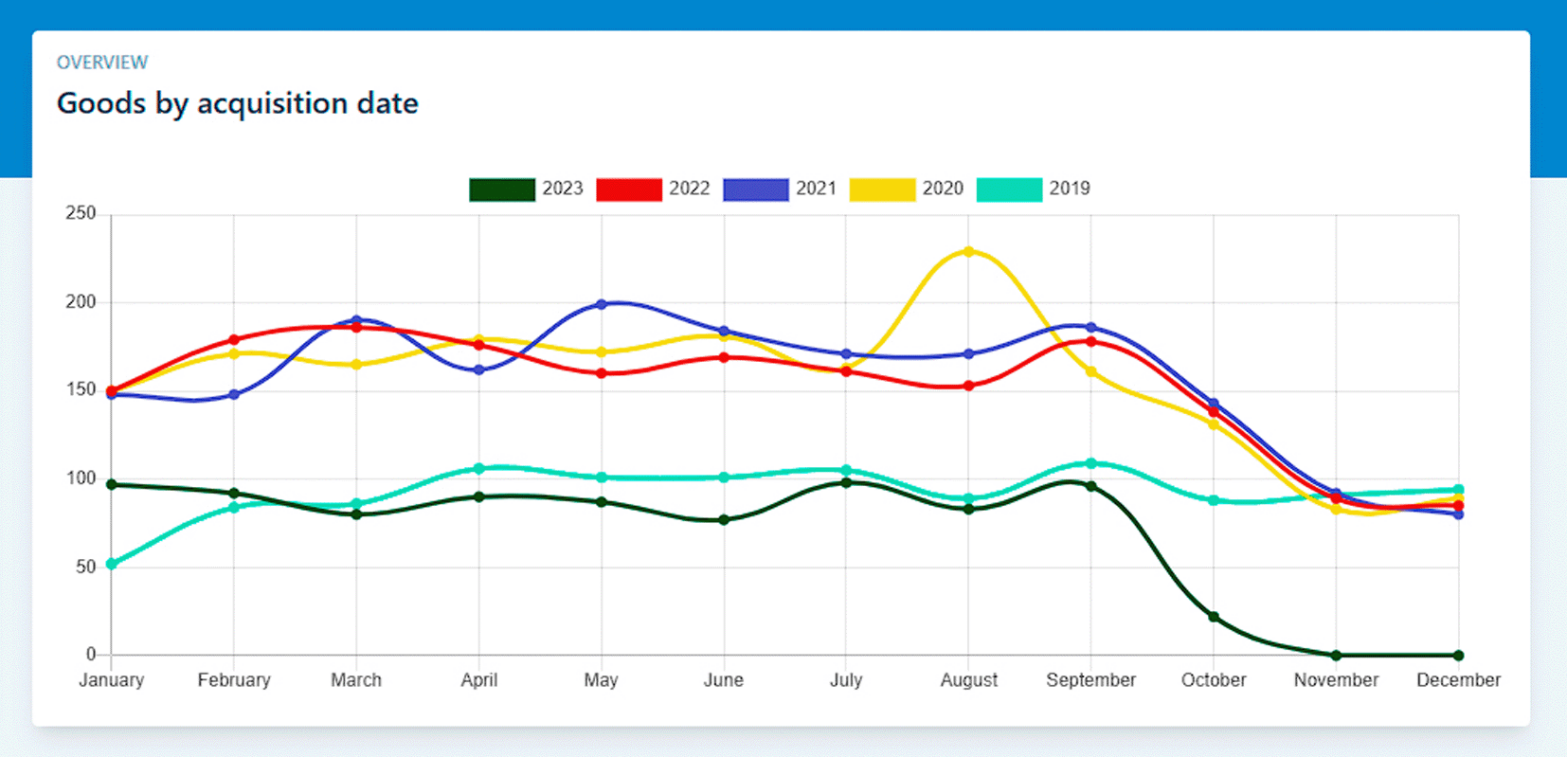
Statistical reports.

In this scenario, we demonstrate how the software facilitates the generation of statistical reports related to goods. Users can select the type of report they require, such as inventory levels, asset depreciation, or goods distribution by category. The software processes the data and generates comprehensive statistical reports, which can aid in decision-making processes and provide insights into the institution’s goods management, as show in the Figure 3.

**Input:**

• Access the “Dashboard” view

**Output:**

• Statistical Report “Goods by acquisition date”

### Use case 3: Report generation according to SBN guidelines

**Figure 4.**
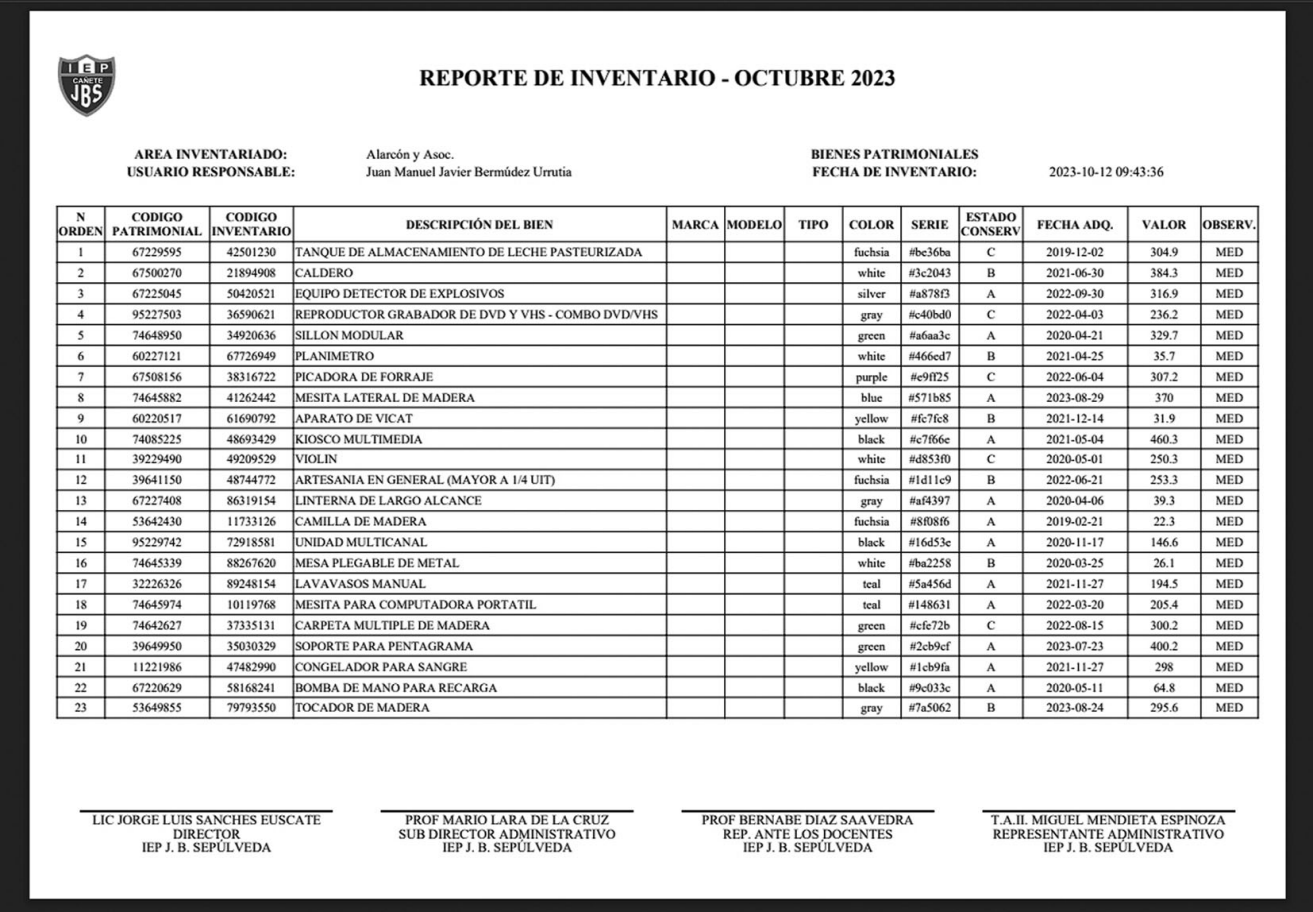
Report generation according to Superintendencia Nacional de Bienes Estatales (SBN) guidelines.

In this scenario, we demonstrate the software’s capability to generate reports that strictly adhere to the guidelines established by the SBN. Users can access the Report menu, where they have the option to generate reports based on specific areas or encompassing all areas. The software will compile and format the necessary data to produce reports that precisely meet SBN standards. This functionality ensures that the institution can efficiently fulfill its reporting obligations to regulatory authorities, as shown in Figure 4.

**Input:**

• Access the “Reports” view
• Select the “Inventory Area”
• Click in “Generate Report” button

**Output:**

• SBN Good Report (PDF)
  • Report Date: Current date

These use cases demonstrate how the software enhances goods management and reporting processes within an educational institution, offering valuable tools for inventory control and compliance with regulatory requirements.

## Discussion

The implementation of the web-based system resulted in a substantial enhancement of the inventory control process, significantly optimizing goods and inventory management. As a result of our software implementation, staff had the opportunity to redirect their efforts toward other administrative or teaching tasks that could enhance the educational institution’s overall performance. This is in line with ( Chancasanampa-Mandujano *et al.*, 2019), where a warehouse management system was used to reduce the stock of raw materials, thus obtaining a more accurate inventory of inputs. In the same way ( Ramos, 2016), reported improved telematics office operations with the implementation of the ISO 9126 standard, resulting in centralized and comprehensive information management. On the other hand, ( Chila & Susi, 2019), highlighted that the management of supply inventory in radiology is one of the main causes of wasted time, with a potential negative impact on patient care in interventional radiology. Therefore, using Lean principles, we evaluated an outdated process and implemented an inventory control system with good results, saving time and money. In turn, these results agree with ( Tejesh & Neeraja, 2018), where a warehouse inventory control system was developed based on IOT (Internet of Things) architecture, which was designed to track products using their corresponding tags and timestamps for further analysis and accurate verification. The developed warehouse inventory management system was efficient, as it was able to perform real-time search operations from the database and update information with the help of web servers.

The enhancement in goods search and localization further streamlined the identification and localization of educational institution goods. This is in agreement with ( López & Pérez, 2016) who obtained an improvement in the search of technical files thanks to the implementation of the computer system, as well as an improvement in the process of patient care. Similarly ( Chiroque, 2018), witnessed changes that greatly benefited institution staff, with goods search, filtering, and reporting processes improving. This work introduced an automated solution to goods management issues, ultimately leading to enhanced inventory management in the institution ( Nemeshaev & Fatkullina, 2021), who studied the processes of collecting, recording and analysing data on their inventory system, found that the pilot implementation of the inventory system improved equipment monitoring, reduced labour costs and the number of errors in the preparation of inventory lists. Similarly, the findings of Ho *et al. * (2021) who developed a blockchain-based system operating under a decentralised accounting mechanism, improved the quality of traceability data and the exchange of reliable information within the spare parts supply chain.

The accuracy and completeness of information regarding educational institution goods significantly improved. The data now remains error-free, unaffected by process distortions, and exhibits consistency between physical and virtual records. Additionally, the software has introduced secure data access mechanisms, granting or restricting personnel access based on their designated roles as administrators or managers. This is in line with ( Fajardo & Lorenzo, 2017), who managed to highlight the result of obtaining truthful information to maintain the optimal inventory, thus achieving optimal and accurate customer service, all as a result of the implementation of the web system. Similarly ( Bonett *et al.*, 2019), reported a reduction in inventory loss due to stock-outs and increased inventory service levels. Their primary objective was to address stock-out issues in SMEs, which previously lacked such effective tools. Similarly Praveen *et al.* (2019), show that the development of a model using artificial neural networks to accurately predict demand contributed to sudden customer demand and minimised the mismatch between supply, demand and associated costs, resulting in increased profit margins. In turn, this is in line with ( De Giovanni, 2021), where the implementation of an intelligent application allows the supply chain to increase sales and improve the production rate, as more consistent and reliable information was obtained from the managed inventory.

Effective reporting saw significant improvement, facilitating better communication between the educational institution and UGEL 08 Cañete, thereby aiding the objectives of both entities. Moreover, it furnishes UGEL 08 Cañete with precise and high-quality information to make informed organizational decisions with the educational institution’s resources. This is in line with ( Rosas & Valecilla, 2019), who pointed out that after the phase following the implementation of a web system in inventory management, operations were optimised in terms of response times for receiving reports and monitoring administrative processes. Similarly ( Guevara, 2019), claimed that, after the development of an inventory management system, it was possible to control materials in a more orderly manner, providing timely and reliable reports for correct and appropriate decisions. The results showed a high degree of agreement with the research of ( Dennert *et al.*, 2021), where it was demonstrated that the use of a universal inventory system allows the rapid generation of physical inventory reports, which in turn preserves the quality of the sample by reducing redundancy and location time. Inventory information was presented in a more user-friendly manner, allowing it to be easily analysed for statistically significant trends, samples had reliable traceability and data was checked for accuracy.

## Conclusions

The web system has significantly improved the inventory control process at the José Buenaventura Fernández Public Educational Institution. After its implementation, the software demonstrated notable enhancements in the three dimensions proposed (search for goods, inventory coverage, and inventory reports). These improvements have greatly benefited the school by optimizing the inventory control process.

Finally, it is recommended that this research be applied in other schools, both public and private, in order to improve and have access to accurate and rapid information on the movable and immovable goods of the institution, since the SBN regulations are applied. At the same time, it is recommended to extend the methods with QR code or barcode, in order to optimise the time of the inventory, and at the same time, this functionality should communicate with the web system of the inventory control process and, for a future stage, consider a connection with the National Goods System of the Ministry of Education.

## Data Availability

The data supporting this research can be found in the “app/database/seedes/data” folder of this repository under the filename “goods-data.csv.” Additionally, a snapshot of the data is archived and available on Zenodo under the following DOI: https://doi.org/10.5281/zenodo.10041267 ( Ramos-Miller (b), 2023). Data are available under the terms of the Creative Commons Attribution 4.0 International license (CC-BY 4.0).

## Software availability

Ramos, M. y Pacheco, A. Towards Inventory Control Excellence: An innovative approach based on a web-based platform.

• Source code available from:
  ○ Backend: https://github.com/mmartinrm97/api-sepulveda/releases/
  ○ Frontend: https://github.com/mmartinrm97/frontend-sepulveda-notus/releases/
• Archived source code at time of publication:
  ○ Backend: https://doi.org/10.5281/zenodo.10041267 ( Ramos-Miller (b), 2023)
  ○ Frontend: https://doi.org/10.5281/zenodo.10023279 ( Ramos-Miller (a), 2023)
• License: MIT License
