# Supplementary material for: Towards inventory control excellence: An innovative approach based on a web-based platform
Source: F1000Res. 2023 Dec 15;12:1471. Originally published 2023 Nov 15. [Version 2] doi: 10.12688/f1000research.140745.2 (PMC10904949; doi:10.12688/f1000research.140745.2)
Supplement: Peer review history

# Reviewer report: Reviewer response for version 2

**DOI:** 10.5256/f1000research.160059.r230727

**Published:** 2024-01-22

**Authors:** Luis de los Santos Valladares

**Affiliations:** 1 University of Cambridge, Cambridge, England, UK

The authors have corrected the manuscript properly and no more corrections are needed.

Are the conclusions about the tool and its performance adequately supported by the findings presented in the article?

Yes

Is the rationale for developing the new software tool clearly explained?

Yes

Is the description of the software tool technically sound?

Yes

Are sufficient details of the code, methods and analysis (if applicable) provided to allow replication of the software development and its use by others?

Yes

Is sufficient information provided to allow interpretation of the expected output datasets and any results generated using the tool?

Yes

Reviewer Expertise:

NA

I confirm that I have read this submission and believe that I have an appropriate level of expertise to confirm that it is of an acceptable scientific standard.

# Reviewer report: Reviewer response for version 1

**DOI:** 10.5256/f1000research.154132.r224020

**Published:** 2024-02-27

**Authors:** Andy Reyes

**Affiliations:** 1 IT Department, Open Nova Lima Perú, Lima, Peru

The research methodology is very well applied.

 The scope is delimited to the educational sector and the research objectives are limited to them, maintaining correct traceability of the research elements.

 The project modules are focused on use cases, although it is a traditional approach, it meets what is necessary to facilitate the design, construction, implementation and maintenance of the proposed system for inventory management in this research.

Are the conclusions about the tool and its performance adequately supported by the findings presented in the article?

Yes

Is the rationale for developing the new software tool clearly explained?

Yes

Is the description of the software tool technically sound?

Yes

Are sufficient details of the code, methods and analysis (if applicable) provided to allow replication of the software development and its use by others?

Yes

Is sufficient information provided to allow interpretation of the expected output datasets and any results generated using the tool?

Yes

Reviewer Expertise:

Red Hat Certified Architect - RHCA, Linux, OpenSource, Software Development, IaaS, PaaS, SaaS, CaaS, IT Automation

I confirm that I have read this submission and believe that I have an appropriate level of expertise to confirm that it is of an acceptable scientific standard.

# Reviewer report: Reviewer response for version 1

**DOI:** 10.5256/f1000research.154132.r224015

**Published:** 2023-11-28

**Authors:** Luis de los Santos Valladares

**Affiliations:** 1 University of Cambridge, Cambridge, England, UK

The authors highlight the importance of inventory management in educational institutions and the challenges associated with it. The work reflects a careful and thoughtful approach to optimising the management of educational facilities through information technology. The decision to use information technology to improve inventory processes demonstrates a dynamic response to current needs.

 However, before accepting the manuscript for indexing, the authors should provide more details on the specific information technologies used during implementation, as well as the evaluation criteria used during system review. This could enhance understanding of the effectiveness and applicability of the system developed.

Are the conclusions about the tool and its performance adequately supported by the findings presented in the article?

Yes

Is the rationale for developing the new software tool clearly explained?

Yes

Is the description of the software tool technically sound?

Yes

Are sufficient details of the code, methods and analysis (if applicable) provided to allow replication of the software development and its use by others?

Yes

Is sufficient information provided to allow interpretation of the expected output datasets and any results generated using the tool?

Yes

Reviewer Expertise:

Data processing

I confirm that I have read this submission and believe that I have an appropriate level of expertise to confirm that it is of an acceptable scientific standard, however I have significant reservations, as outlined above.

# Response

**Published:** 2023-12-11

**Authors:** Alex Pacheco

**Affiliations:** Universidad Nacional de Cañete, Peru

This new version provides more details on the specific information technologies used during implementation, as well as the evaluation criteria used during the system review. Information on development technologies, strategic plug-in integration, technological details for indexation consideration and evaluation criteria during system review has been added.
